# Compositional Brain Age Profiles Reveal Disease-Related Neuroimaging Patterns Beyond the Brain Age Gap

**DOI:** 10.3390/biology15211905

**Published:** 2026-10-22

**Authors:** Wei Wang, Yihang Xiao, Jiajie Peng

**Affiliations:** 1 AI for Science Interdisciplinary Research Center, School of Computer Science, Northwestern Polytechnical University, Xi’an 710072, China; x904940151@mail.nwpu.edu.cn (Y.X.); jiajiepeng@nwpu.edu.cn (J.P.); 2 Key Laboratory of Big Data Storage and Management, Northwestern Polytechnical University, Ministry of Industry and Information Technology, Xi’an 710072, China

**Keywords:** brain age, brain age gap, multimodal magnetic resonance imaging, neurological disorders, neuroimaging heterogeneity, UK Biobank

## Abstract

The brain age gap (BAG)—the difference between the predicted and chronological age—does not describe the imaging patterns underlying such deviation. To characterize these patterns, we developed a semantic-guided mixture-of-experts model using multimodal imaging-derived phenotypes and their metadata. Global and local expert outputs were combined to predict age and retained as an individualized composition profile. An evaluation in 37,894 UK Biobank participants used separate reference and clinical cohorts, with a test mean absolute error of 2.784–2.811 years. Participants with nearly identical standardized BAGs had different profiles, indicating imaging heterogeneity at similar levels of age deviation. Adding the profile to a baseline of age, standardized BAG, sex, and imaging center improved discrimination in four disease contrasts. The area under the receiver operating characteristic curve (AUC) increased by 0.018–0.060, with gains persisting after matching by age and standardized BAG. Higher AUC point estimates than equal-dimensional controls suggested that adding dimensions alone did not explain the gains. Combining the age-trained profile with unsupervised principal components improved discrimination further, suggesting complementary imaging information. These findings support composition profiles for examining disease-related imaging differences beyond overall brain age deviation.

## Simple Summary

Brain age models estimate how old a person’s brain appears from magnetic resonance imaging, but the usual output is a single number that cannot indicate which imaging patterns produced the estimate. We developed a method that uses thousands of measurements from several brain imaging modalities and organizes them into multiple learned patterns of brain ageing. For each participant, the method produces both an estimated brain age and an individualized profile describing the combination of imaging patterns underlying the estimate. In 37,894 UK Biobank participants, the model predicted age accurately and produced stable imaging pattern organization. Importantly, people with almost identical overall brain age differences could still have very different composition profiles. These profiles also helped to distinguish between selected neurological disease groups even after participants were matched by age and overall brain age difference. The results suggest that brain ageing may be better understood not only by asking how much a brain differs from its expected age but also by examining the combination of imaging patterns associated with this difference.

## 1. Introduction

Biological age measures aim to characterize variation in ageing that is not captured by chronological age alone. In neuroimaging, brain age models operationalize this concept by predicting the chronological age from magnetic resonance imaging (MRI)-derived measurements and comparing the predicted age with the observed age [1,2]. The resulting brain age gap (BAG) is typically interpreted as the extent to which an individual’s brain appears older or younger than expected for their chronological age [3,4]. Larger positive BAG values, reflecting accelerated brain ageing, have been associated with neurological and psychiatric disorders, cognitive decline, and adverse health outcomes [5,6,7]. These associations have established the BAG as an increasingly used marker for studying age-related brain health and disease vulnerability [4,8].

Although the BAG provides a concise summary of brain age deviation, it does not retain the compositional information encoded in the underlying high-dimensional imaging features. A single BAG value cannot reflect whether similar brain age deviations reflect comparable or distinct underlying neuroimaging patterns. This distinction is important because brain ageing is expressed through heterogeneous patterns across cortical, subcortical, white matter, functional connectivity, and global MRI phenotypes, which may contribute differently to the same overall brain age deviation [9,10].

Recent multimodal brain age studies have shown that structural, diffusion, and functional MRI provide complementary information for characterizing brain ageing and can support modality-specific or integrated brain age estimates [8,11,12,13]. Regional and explainable brain age approaches have further localized age-related deviations and identified anatomical features contributing to individual predictions [14,15,16]. In parallel, data-driven studies have identified multiple structural and functional ageing patterns and assigned individuals scores along distinct ageing-related dimensions [17,18,19]. Together, these studies indicate that brain ageing is multidimensional and can be characterized across modalities, regions, and distributed ageing patterns.

Nevertheless, existing multimodal, regional, and dimensional approaches typically characterize this heterogeneity through modality- or region-specific estimates, post hoc explanations of scalar predictions, or separate ageing dimensions. Less explored is whether multiple imaging patterns can be jointly learned within a unified brain age model and expressed as participant-specific contributions that together form an interpretable composition of the predicted brain age.

Here, we developed a semantic-guided mixture-of-experts framework for individualized brain age composition profiling using multimodal imaging-derived phenotypes (IDPs). The framework uses semantic information associated with the IDPs to organize heterogeneous imaging phenotypes across multiple specialized experts, encouraging specialized experts to capture distinct ageing-related imaging patterns. The expert-specific representations are then integrated to estimate the brain age and jointly form an individualized brain age composition profile that characterizes their relative contributions to the prediction.

## 2. Materials and Methods

### 2.1. Study Population and Multimodal IDPs

**Figure 1:**
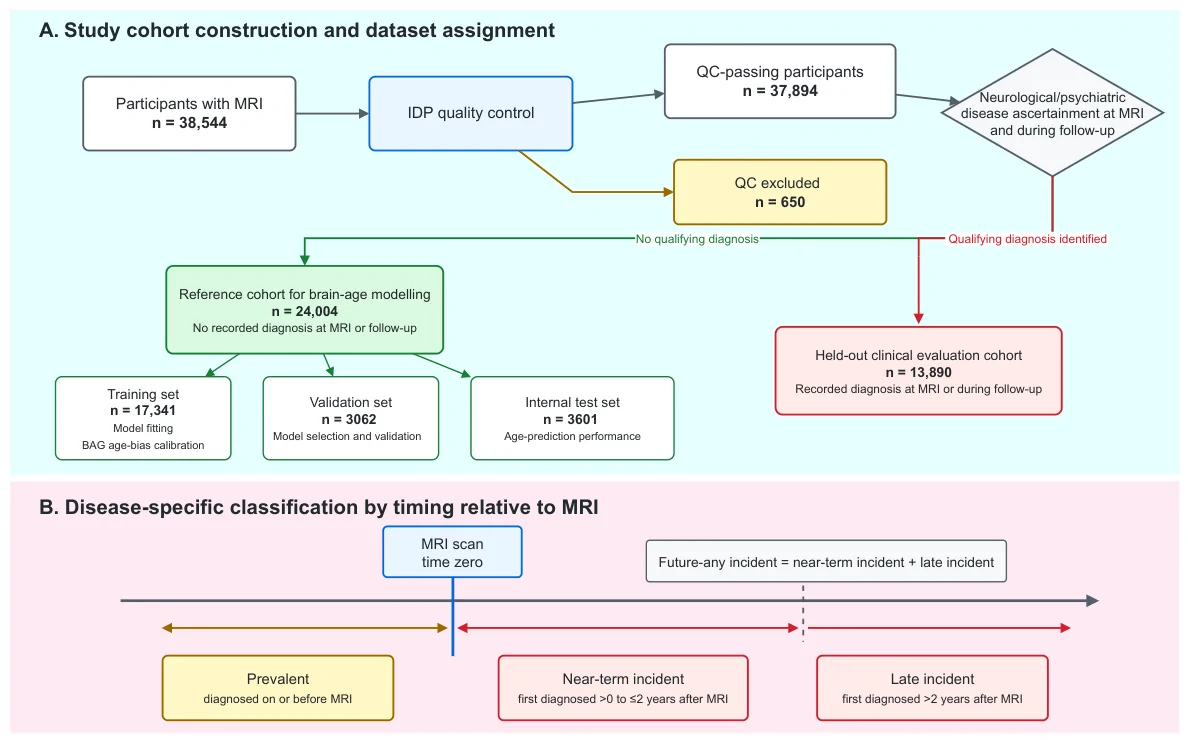
Study cohort construction and disease-specific timing classification. (**A**) Cohort construction and analysis roles. Participants were assigned to the reference cohort or clinical evaluation cohort after the quality control of imaging-derived phenotypes (IDPs) and review of qualifying neurological and psychiatric disease records. The reference cohort was further divided into training, validation, and test sets, whereas participants with qualifying diagnoses were reserved for clinical evaluation. (**B**) Disease-specific timing classification relative to magnetic resonance imaging (MRI). The brown arrow indicates prevalent disease, whereas the two red arrows indicate the incident categories comprising future-any incident disease. For each disease, cases were classified as prevalent if the first qualifying record occurred on or before MRI, near-term incident if it occurred >0 to ≤2 years after MRI, and late incident if it occurred >2 years after MRI. Future-any incident combined the near-term and late incident categories. Abbreviations: BAG, brain age gap; IDP, imaging-derived phenotype; MRI, magnetic resonance imaging; QC, quality control.

We analyzed multimodal IDPs from UK Biobank MRI data. Cohort construction and analysis roles are summarized in Figure 1A. IDPs were generated by the standardized UK Biobank image processing pipeline, including modality-specific preprocessing, registration, anatomical alignment, segmentation, and phenotype extraction [9]. They covered structural MRI, diffusion MRI, resting-state and task-based functional MRI, susceptibility-weighted imaging, and T2-FLAIR imaging. Each IDP was accompanied by metadata describing its anatomical and imaging context. Whole-brain or hemispheric summary measures were designated as global IDPs; the remaining regional and distributed measures were designated as local IDPs.

The model development reference cohort included participants with no qualifying records for prespecified neurological or psychiatric conditions at or before MRI or during the available follow-up. Exclusions were based on ICD-10 definitions and UK Biobank disease records and covered dementia; cerebrovascular disease; epilepsy; migraine and other headache syndromes; and selected inflammatory, degenerative, and demyelinating neurological disorders, as well as substance-related, psychotic, and mood disorders. Other diseases did not preclude eligibility. The exclusion criteria and ICD-10 code ranges are listed in Table A1 (Appendix A). The reference cohort was randomly divided into training, validation, and test sets, whereas participants with prevalent or incident qualifying diagnoses were reserved for clinical evaluation.

Disease timing was assigned separately for each disease using the first qualifying record relative to MRI (Figure 1B). Prevalent disease was recorded at or before MRI. Near-term incident disease was first recorded >0 to ≤2 years after MRI; late incident disease was first recorded >2 years after MRI. Future-any incident disease combined both incident categories within the available follow-up. Participants without a qualifying record were not cases for that disease. Because participants could have multiple diseases, the same participant could be assigned to different temporal categories for different diseases. Follow-up ended at the earliest point among death, recorded loss to follow-up, or administrative censoring.

Participants were excluded if more than 20% of their IDPs were missing. Preprocessing parameters were estimated from the brain age training set only and applied unchanged to the validation, test, and clinical evaluation cohorts. For each IDP, missing values were temporarily imputed with the median of that IDP calculated from the training set. The temporarily imputed training data were then used to estimate feature-wise means and standard deviations for z-score standardization. After standardization, originally missing entries were reset to zero, while their missing status was retained as a separate binary indicator.

### 2.2. Overview of the Semantic-Guided Mixture-of-Experts Framework

**Figure 2:**
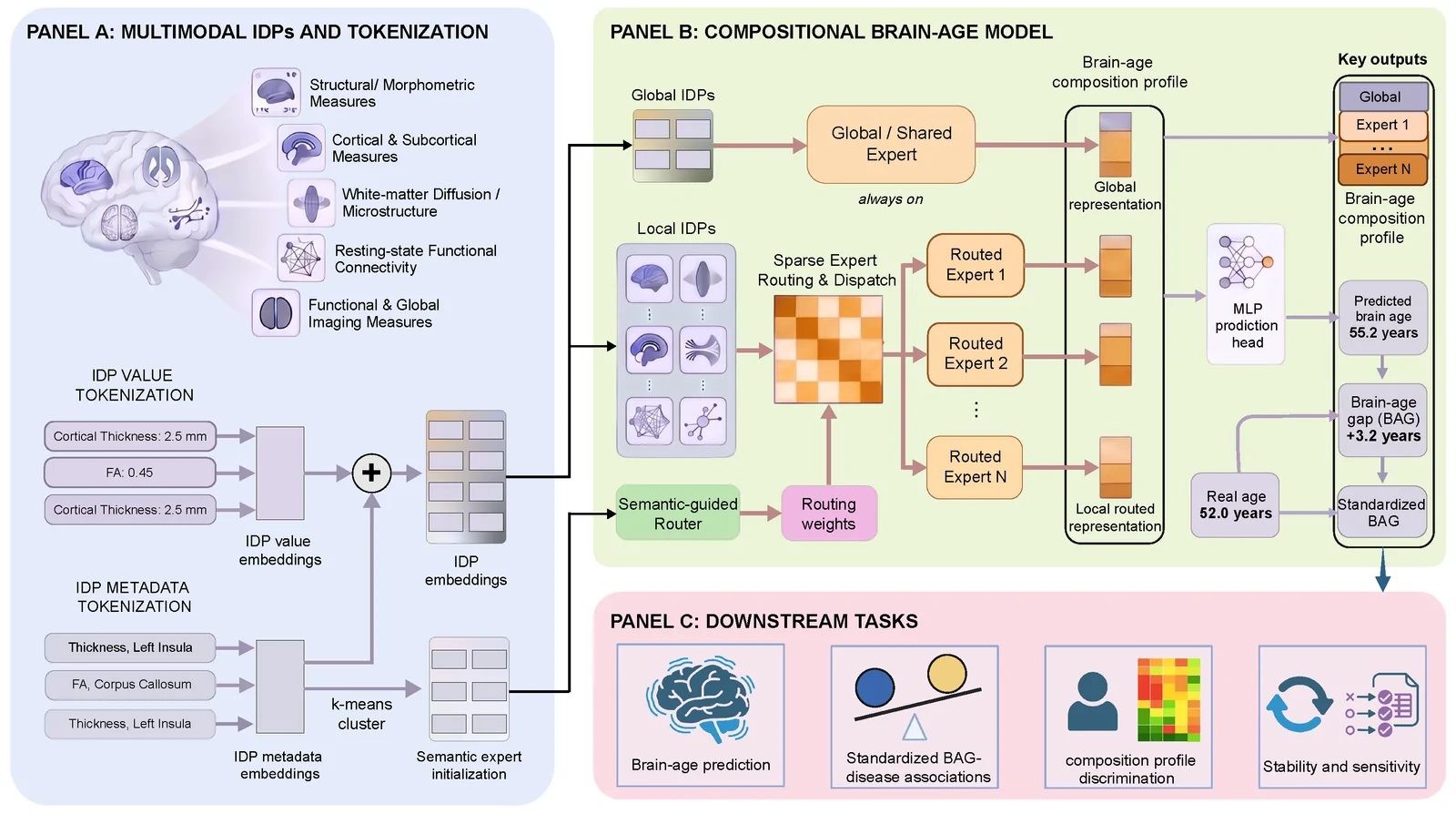
Overview of the proposed semantic-guided mixture-of-experts framework. (**A**) Participant-specific measurements and semantic metadata are combined into imaging-derived phenotype (IDP) representations. Metadata clustering initializes local expert routing. (**B**) Global and sparsely routed local experts produce outputs that form the composition profile. A multilayer perceptron (MLP) maps this profile to brain age. The predicted-minus-chronological age difference undergoes age bias correction and standardization to obtain a standardized brain age gap (BAG). (**C**) Model outputs are evaluated for brain age prediction, standardized BAG–disease associations, disease-related differences in composition profiles, and robustness through stability and sensitivity analyses. Arrow colors distinguish stages of the workflow: light purple denotes embedding construction and output derivation, black denotes the transfer of representations from panel (**A**) to panel (**B**), muted red denotes routing and expert processing, and blue denotes the transfer of model outputs to downstream tasks. Abbreviations: BAG, brain age gap; IDP, imaging-derived phenotype; MLP, multilayer perceptron.

The framework used multimodal IDPs to predict brain age while retaining expert-specific representations for each participant (Figure 2). Each participant-specific IDP representation combined an encoded measurement with a metadata-derived semantic embedding of that IDP. Global IDPs were processed by an always-active global expert, whereas local IDPs were sparsely assigned to local experts according to metadata-guided routing. The resulting global and local expert representations formed a participant-specific composition profile, which was used by the prediction head to estimate brain age.

### 2.3. Joint Representation of IDP Measurements and Semantic Metadata

For a given participant, $x_{i}$ denotes the standardized value of IDP *i*, and $\delta_{i}$ denotes its missingness indicator, with $\delta_{i}=1$ for missing values and $\delta_{i}=0$ for observed values. A shared value encoder mapped $\left(x_{i},\;\delta_{i}\right)$ to a value embedding $e_{i}^{\mathrm{val}}$. The metadata describing each IDP were encoded using a frozen BioLORD [20] encoder to obtain a pretrained semantic embedding $e_{i}^{\mathrm{meta}}$. A trainable metadata encoder used a shared backbone and separate projections to produce ${\tilde{e}}_{i}^{\mathrm{meta}}$ for value fusion and $r_{i}$ for expert routing. The fusion embedding matched the dimension of the value embedding, allowing element-wise addition:

(1) $$z_{i}=e_{i}^{\mathrm{val}}+{\tilde{e}}_{i}^{\mathrm{meta}}$$

The resulting IDP embedding combined participant-specific measurements with semantic information shared across participants. The metadata encoder was trained jointly with the brain age model, while the BioLORD embeddings remained fixed.

### 2.4. Semantic-Guided Mixture-of-Experts Brain Age Modeling

#### 2.4.1. Semantic Initialization of Expert Specialization

Before brain age training, the pretrained BioLORD metadata embeddings of local IDPs were clustered using *k*-means to initialize the IDP-to-expert assignments. We denote the number of local routed experts by *K*. The *K* clusters defined the initial assignments to the corresponding local experts. This initialization provided a semantic starting point for routing, grouping related imaging phenotypes while allowing their assignments to be refined during model optimization. Global IDPs were excluded from clustering and processed by the global expert.

#### 2.4.2. Mixture-of-Experts Learning for Heterogeneous Ageing Patterns

During brain age training, the router mapped the metadata-derived representation $r_{i}$ of each local IDP *i* to routing scores over the *K* routed experts,

(2) $$s_{i}=R\left(r_{i}\right),\;s_{i}\in R^{K},$$

where R(·) denotes the learnable router and $s_{ik}$ is the routing score of IDP *i* for expert *k*. Because routing was based only on phenotype metadata, these scores were shared across participants and did not depend on participant-specific IDP values.

Sparse routing retained the experts with the highest scores for each IDP,

(3) $$T_{i}=\mathrm{TopX}\left(s_{i}\right),$$

where $T_{i}$ denotes the set of experts selected for IDP *i*, and *X* is the number of selected experts, set to 2 in this study. Routing weights were normalized across the selected experts,

(4) $$\alpha_{ik}=\frac{\exp(s_{ik})}{\sum_{j\in T_{i}}\exp\left(s_{ij}\right)},\;k\in T_{i},$$

with $\alpha_{ik}=0$ for experts not selected for IDP *i*.

The set of IDPs assigned to routed expert *k* was therefore

(5) $$I_{k}=\left\{i|k\in T_{i}\right\}.$$

For a given participant, each routed expert aggregated the corresponding IDP representations and generated a scalar expert-specific representation,

(6) $$h_{k}=E_{k}\;[\mathrm{Agg}\{\alpha_{ik}z_{i}:i\in I_{k}\}],\;k=1,\ldots ,K.$$

Here, $z_{i}$ is the IDP embedding defined above, Agg(·) denotes aggregation over the routed IDPs, and $E_{k}\left(\cdot \right)$ denotes the transformation of local expert *k*, which produces the scalar output $h_{k}$. The original missingness mask was applied during aggregation so that missing IDP measurements did not contribute to the corresponding expert representation. Because an IDP could be routed to more than one expert, the resulting expert-specific IDP subsets could partially overlap.

#### 2.4.3. Incorporating Global Ageing Signals

Let G denote the set of global IDPs defined above. For a given participant, the scalar global representation was obtained as

(7) $$h_{g}=E_{g}\;[\mathrm{Agg}\{z_{i}:i\in G\}].$$

Here, Agg(·) denotes aggregation over the global IDPs, and $E_{g}\left(\cdot \right)$ denotes the global expert transformation producing the scalar output $h_{g}$. The global representation was concatenated with the *K* local expert-specific representations to form the brain age composition profile,

(8) $$p=\left(h_{g},h_{1},\ldots ,h_{K}\right),\;p\in R^{K+1}.$$

With K=10 local experts and one global expert, the profile contained 11 scalar components.

### 2.5. Brain Age Prediction and Optimization Objective

The composition profile p was passed to a multilayer perceptron (MLP) prediction head to estimate the brain age, expressed in years:

(9) $$\hat{y}=\mathrm{MLP}\left(p\right)$$

On the original age scale, the age prediction objective was the mean squared error between the predicted brain age and chronological age,

(10) $$L_{\mathrm{age}}=\frac{1}{N}\sum_{n=1}^{N}{\left({\hat{y}}_{n}-y_{n}\right)}^{2}.$$

Here, *N* denotes the number of training participants. For participant *n*, ${\hat{y}}_{n}$ is the predicted brain age and $y_{n}$ is the chronological age at MRI, both expressed in years. The IDP encoder, routed experts, metadata-conditioned router, global expert, and prediction head were optimized jointly during brain age training.

### 2.6. Evaluation and Downstream Analyses

#### 2.6.1. Brain Age Prediction and Model Selection

Brain age prediction performance was evaluated using the mean absolute error (MAE), expressed in years. The number of local experts was selected by grid search over K=6–12. We selected K=10 because it achieved the lowest validation MAE, and we fixed this configuration before evaluation on the test set. Models were trained using three random seeds (2026, 42, and 3407), with identical cohort splits across seeds. The test set was used for final brain age prediction evaluation, and the held-out clinical evaluation cohort was used for downstream disease analyses. Neither was used for brain age model selection.

#### 2.6.2. Brain Age Gap Standardization

The raw BAG was the difference between the predicted brain age and chronological age, both expressed in years:

(11) $${\mathrm{BAG}}_{n}^{\mathrm{raw}}={\hat{y}}_{n}-y_{n}.$$

The raw BAG can retain a systematic age dependence arising from prediction bias. We regressed it on chronological age in the training reference cohort $C_{\mathrm{train}}$. The fitted intercept ${\hat{\gamma }}_{0,\mathrm{train}}$ and slope ${\hat{\gamma }}_{1,\mathrm{train}}$ were used to remove the linear age trend:

(12) $${\mathrm{BAG}}_{n}^{\mathrm{corrected}}={\mathrm{BAG}}_{n}^{\mathrm{raw}}-\left({\hat{\gamma }}_{0,\mathrm{train}}+{\hat{\gamma }}_{1,\mathrm{train}}y_{n}\right).$$

The corrected BAG remains in years and measures the deviation from the training cohort’s fitted age-specific expectation.

We then standardized the corrected BAG by its training-reference standard deviation (SD):

(13) $${\mathrm{BAG}}_{n}^{\mathrm{standardized}}=\frac{{\mathrm{BAG}}_{n}^{\mathrm{corrected}}}{{\mathrm{SD}}_{\mathrm{BAG},\mathrm{train}}}.$$

Here, ${\mathrm{SD}}_{\mathrm{BAG},\mathrm{train}}$ is the SD of the corrected BAG in the training reference cohort. The standardized BAG is dimensionless; a value of +1 indicates a positive deviation of one training reference SD. All subsequent analyses used this standardized scale.

The correction parameters and residual SD were estimated separately for each model seed using the training reference cohort only. They were applied unchanged to the validation, test, and clinical evaluation cohorts.

#### 2.6.3. Composition Profile Characterization

Expert organization was characterized by the number and phenotype composition of assigned IDPs. Agreement between expert assignments across model seeds was quantified using the adjusted Rand index (ARI) and normalized mutual information (NMI).

Associations between the standardized BAG and each expert representation were summarized using the coefficient of determination ($R^{2}$). Participants were sorted by standardized BAG and grouped into non-overlapping adjacent pairs. For each pair, we calculated the absolute standardized BAG difference and the Euclidean distance between the complete 11-dimensional composition profiles. Spearman’s correlation assessed the association between these two quantities.

#### 2.6.4. Disease Association Analysis

A separate linear regression compared cases with reference-cohort participants for each disease and temporal phase. Disease status was coded as $D_{n}=1$ for cases and $D_{n}=0$ for reference participants:

(14) $${\mathrm{BAG}}_{n}^{\mathrm{standardized}}=\beta_{0}+\beta_{1}D_{n}+\beta_{\mathrm{cov}}^{⊤}c_{n}+\varepsilon_{n}.$$

The covariate vector $c_{n}$ contained MRI age $y_{n}$, sex, and imaging center; $\varepsilon_{n}$ denotes the residual. The fitted coefficient ${\hat{\beta }}_{1}$ quantifies the adjusted mean case–reference difference in training-residual SD units. Positive coefficients indicate a higher standardized BAG in cases than in reference-cohort participants.

Association models were fitted separately for each brain age model seed. Disease coefficient *P* values were adjusted using the Benjamini–Hochberg false discovery rate procedure. Consistent significance required an FDR-adjusted P<0.05 in all three seeds. Effect estimates were summarized across seeds.

#### 2.6.5. Composition Profile Analysis Beyond Standardized BAG

For each disease contrast, participants meeting exactly one of the two target disease definitions were included. Other diagnoses did not preclude inclusion.

All classifiers used L2-regularized logistic regression. The baseline included the MRI age, standardized BAG, sex, and imaging center. For each representation in Table 1, a separate augmented classifier was fitted by adding the representation to the baseline covariates. These representations included PCA-11, PCA-22, Bottleneck-11, Random-MoE-11, the complete 11-dimensional composition profile p, and Profile + PCA-11. Within each contrast, AUCs were evaluated using the same participants and repeated five-fold outer cross-validation splits. For each classifier, the inverse regularization strength *C* was selected by inner cross-validation within each outer training fold. All classifiers used the same candidate set, $C\in \{10^{-3},10^{-2},10^{-1},1,10,100\}$, and selection procedure.

Control representations were constructed using the training reference cohort and frozen before clinical evaluation. PCA-11 and PCA-22 comprised the first 11 and 22 principal components of the IDP data, respectively, and served as unsupervised dimensionality reduction controls. Bottleneck-11 used an 11-dimensional age-supervised representation learned without expert decomposition. Random-MoE-11 retained the expert architecture, routing constraints, age prediction objective, and training budget of the proposed model but randomized the organization of IDPs across experts. The proposed representation comprised the complete 11-dimensional global and local expert profile. Profile + PCA-11 concatenated this profile with PCA-11, yielding 22 features. Classifier-specific preprocessing and regularization selection were performed within the corresponding training folds.

#### 2.6.6. BAG-Matched Sensitivity Analysis

A matched sensitivity analysis further assessed whether the profile retained discriminative information among participants with similar standardized BAGs. Participants were matched one-to-one by MRI age and standardized BAG within sex and imaging center strata. Only the baseline and baseline-plus-profile classifiers were evaluated. All other procedures followed the preceding section, with each matched pair kept in the same validation fold.

Within this matched analysis, matched-pair bootstrap resampling provided 95% confidence intervals for profile-versus-baseline AUC differences separately for each brain age model seed. Identical resamples were used for both classifiers. Evidence was classified as robust when the interval lay above zero in all three seeds. A positive mean gain with an interval including zero in at least one seed was classified as indicative.

## 3. Results

### 3.1. Study Population and Brain Age Model Performance

Of 38,544 participants with MRI, 37,894 passed IDP quality control and 650 were excluded (Figure 1 and Table 2). The reference cohort comprised 24,004 participants and was divided into training (n=17,341), validation (n=3062), and test (n=3601) sets. The clinical evaluation cohort included 13,890 participants with prevalent or incident qualifying diagnoses. These participants were reserved for downstream disease analyses. The mean age was similar across the three reference cohort splits (63.8–63.9 years) and was 64.3 years in the clinical evaluation cohort. Women comprised approximately 51% of each reference cohort split and 57.2% of the clinical evaluation cohort.

Brain age prediction performance was similar across the three random seeds. Validation MAE ranged from 2.682 to 2.732 years, and test MAE ranged from 2.784 to 2.811 years. Across the three seeds, the test root mean squared error (RMSE) ranged from 3.497 to 3.547 years, and $R^{2}$ ranged from 0.783 to 0.789 on the same 3601 reference test participants. These estimates describe prediction within the UK Biobank reference cohort. A single-center training and unseen-center evaluation analysis is reported in Appendix B.

### 3.2. Brain Age Composition Profiles Differed Among Participants with Similar Standardized BAGs

**Figure 3:**
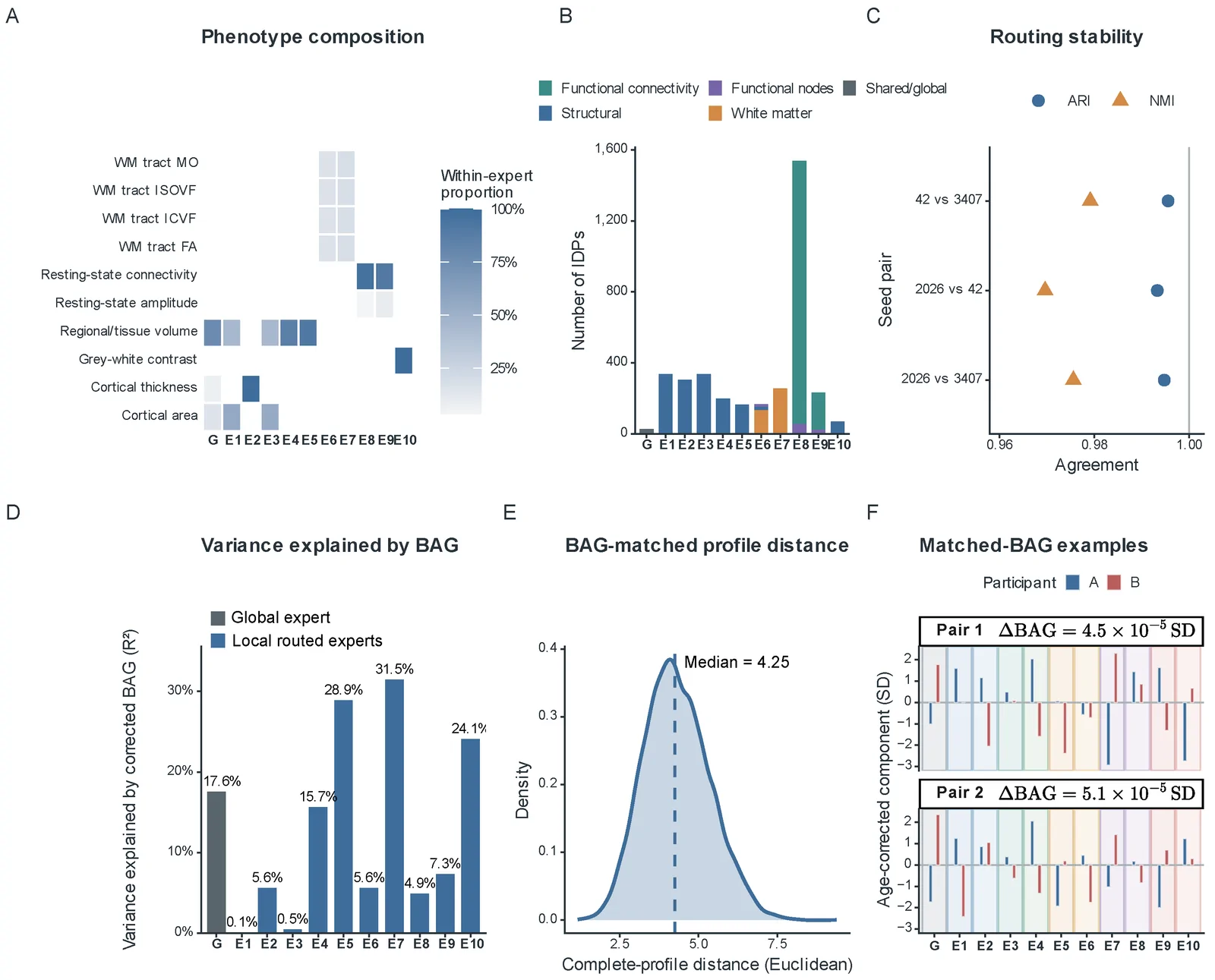
Semantic-guided expert specialization and brain age composition heterogeneity. (**A**) Phenotype composition of the global expert (G) and ten local routed experts (E1–E10). (**B**) Number and modality composition of imaging-derived phenotypes (IDPs) assigned to each expert. (**C**) Routing stability across model seeds measured by adjusted Rand index (ARI) and normalized mutual information (NMI). (**D**) Variance in each expert representation explained by standardized BAG, summarized by the coefficient of determination ($R^{2}$). (**E**) Distribution of composition profile distances among participants with nearly identical standardized BAGs. (**F**) Representative BAG-matched participant pairs showing distinct composition profiles. BAG denotes brain age gap; SD denotes standard deviation. BAG values are expressed in training-residual SD units. Abbreviations: WM, white matter; MO, mode of anisotropy; ISOVF, isotropic volume fraction; ICVF, intracellular volume fraction; FA, fractional anisotropy.

The global expert processed 26 whole-brain or hemispheric IDPs, and ten local routed experts processed the remaining 3604 IDPs (Figure 3A,B). Structural groups differed in their emphasis on cortical area and volume, cortical thickness, regional volume and intensity, and cortical grey–white contrast. Several also distinguished between left- and right-hemisphere measurements. White matter groups mainly contained tract microstructure measures, while functional groups included connectivity and node amplitude phenotypes. Local group sizes ranged from 70 to 1539 IDPs; the largest group was dominated by resting-state functional connectivity. The resulting groups therefore differed in both measurement type and size.

Expert assignments were highly stable across the three model seeds with identical participants and semantic initialization (Figure 3C). The pairwise ARI ranged from 0.993 to 0.996, and the NMI ranged from 0.970 to 0.979.

Associations with the standardized BAG varied in strength across the expert representations (Figure 3D). The expert-wise $R^{2}$ ranged from 0.1% to 31.5%, with an $R^{2}$ of 17.6% for the global representation. In this analysis, the standardized BAG accounted for only part of the variation in each expert representation.

Participants with nearly identical standardized BAGs nevertheless had different composition profiles (Figure 3E,F). Sorting participants by standardized BAG yielded 8745 non-overlapping adjacent pairs with a median absolute BAG difference of 0.00017 SD. Their complete profiles had a median Euclidean distance of 4.25 (interquartile range, 3.58–5.00). Within these BAG-matched pairs, no association was detected between the profile distance and the residual difference in the standardized BAG (Spearman’s ρ=0.003, p=0.790). Figure 3F shows two representative high-distance pairs with distinct global and local expert output patterns. These examples illustrate the variation across expert outputs at a similar level of overall brain age deviation.

### 3.3. Standardized BAG Showed Broad Disease Associations Across Diagnoses and Temporal Phases

**Figure 4:**
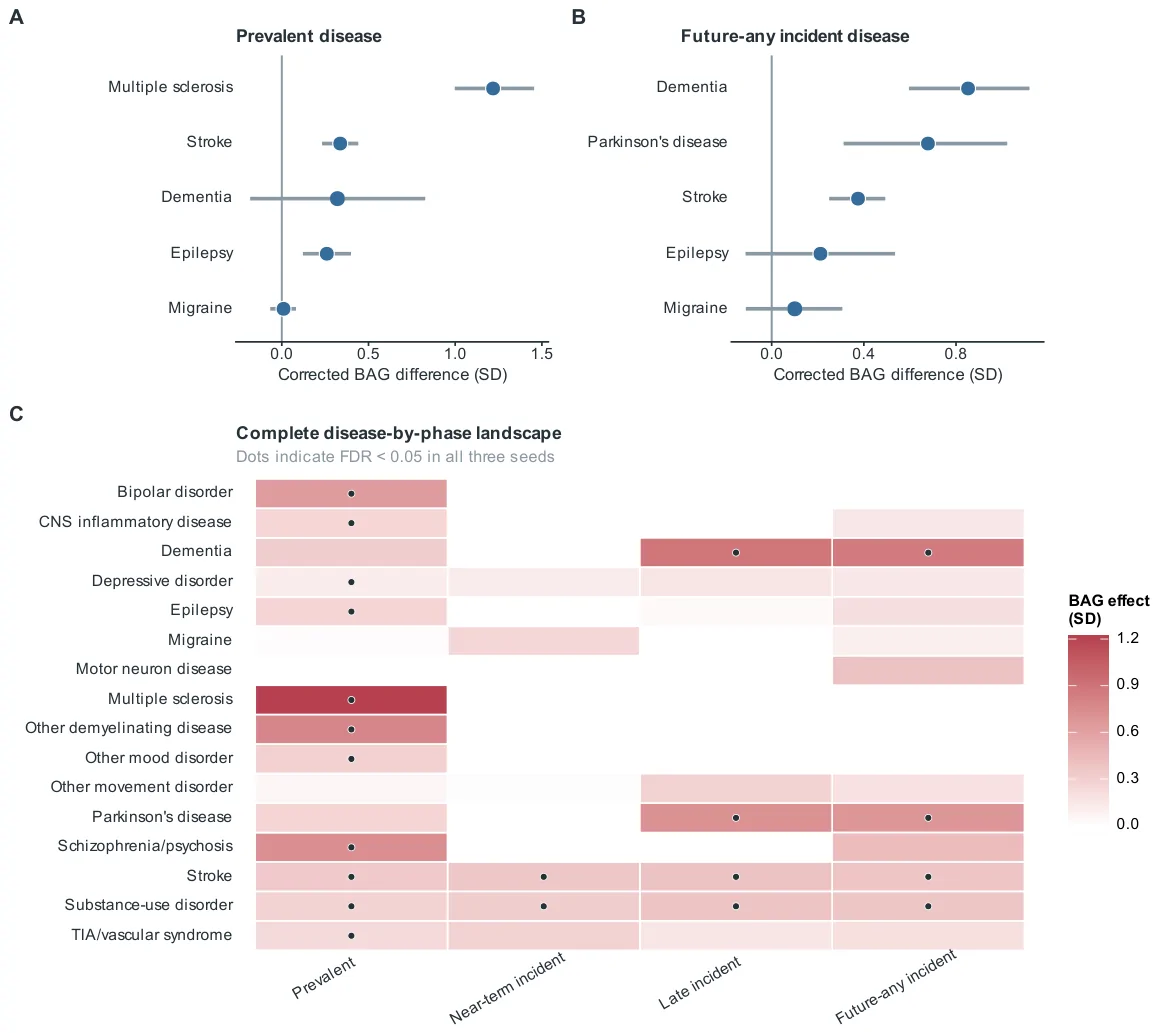
Standardized brain age gap (BAG) associations across diseases and temporal phases. (**A**) Adjusted standardized BAG differences for selected prevalent diagnoses. (**B**) Adjusted standardized BAG differences for selected diagnoses recorded after MRI (future-any incident). (**C**) Complete disease-by-phase landscape of standardized BAG differences for prevalent, near-term incident, late incident, and future-any incident diagnoses. Future-any incident combines near-term and late incident diagnoses. Differences were adjusted for magnetic resonance imaging (MRI) age, sex, and imaging center and are expressed in training-residual standard deviation (SD) units. Dots indicate false discovery rate (FDR)-adjusted p<0.05 in all three model seeds. Abbreviations: TIA, transient ischaemic attack; CNS, central nervous system.

The standardized BAG was higher in several prevalent neurological and psychiatric disease groups than in the reference cohort (Figure 4). The mean adjusted differences across the three model seeds were largest for multiple sclerosis (1.218 training-residual SD units). Other prominent differences were observed for other demyelinating disease (0.773), schizophrenia/psychosis (0.733), and bipolar disorder (0.636). These four associations were FDR-significant in all three seeds. Smaller positive differences were observed for stroke (0.337), epilepsy (0.260), and depressive disorder (0.122), which were also FDR-significant in all seeds. Prevalent migraine showed a small adjusted difference (0.010 SD units) that was not FDR-significant in any seed.

Positive associations were also observed for diagnoses recorded after MRI (future-any incident). The mean adjusted differences across seeds were 0.852 SD units for dementia, 0.678 for Parkinson’s disease, 0.375 for stroke, and 0.359 for substance use disorder. All four associations were FDR-significant in all three seeds. The Parkinson’s disease estimate warrants caution because the group included only 47 cases.

Phase-specific analyses showed positive associations for stroke and substance use disorder in both the near-term and late incident groups (Figure 4C). For stroke, the mean adjusted differences were 0.355 and 0.382 SD units in the near-term and late incident groups, respectively. The corresponding estimates for substance use disorder were 0.323 and 0.379 SD units. All four associations were FDR-significant in all three seeds. Late incident dementia also showed a positive association (0.886 SD units; 77 cases), being FDR-significant in all three seeds. These phase-specific estimates describe differences between separate case groups and the reference cohort. Other disease-by-phase comparisons were non-significant.

### 3.4. Brain Age Composition Profiles Captured Disease-Related Information Beyond Standardized BAG

Adding the composition profile increased the AUC in all four disease contrasts relative to the baseline containing the standardized BAG and demographic covariates (Table 3). The largest gain occurred for future-any dementia versus stroke, where the AUC increased from 0.613 to 0.673 (+0.060). For prevalent multiple sclerosis versus migraine, the AUC increased from 0.709 to 0.759 (+0.050). Smaller gains were observed for prevalent multiple sclerosis versus epilepsy (+0.035) and epilepsy versus migraine (+0.018). These comparisons support additional disease-discriminative information in the profile beyond the baseline covariates.

The profile had higher AUCs than all three 11-dimensional controls in every contrast. Its advantage over PCA-11 ranged from 0.010 to 0.020, while Bottleneck-11 remained close to the baseline performance. The profile also had higher AUCs than PCA-22 in three contrasts. In future-any dementia versus stroke, however, PCA-22 achieved a slightly higher AUC than the profile (0.676 versus 0.673). Thus, the profile’s advantage over alternative representations varied across disease contrasts, despite its consistently higher point estimates than the equal-dimensional controls.

PCA-11 captured the dominant variation in the imaging features without age supervision, whereas the composition profile was learned through the brain age prediction objective. Combining PCA-11 with the composition profile yielded the highest AUC across all four disease contrasts, with values ranging from 0.669 to 0.787. Compared with the profile alone, the combined representation increased the AUC by 0.018–0.042. It also outperformed PCA-22, which matched the combined representation in dimensionality, by 0.024–0.062. These results indicate that the improvement was not explained by increased dimensionality alone and that the two representations captured partly non-overlapping information relevant to disease discrimination.

### 3.5. Composition Profile Gains Persisted in BAG-Matched Disease Comparisons

**Figure 5:**
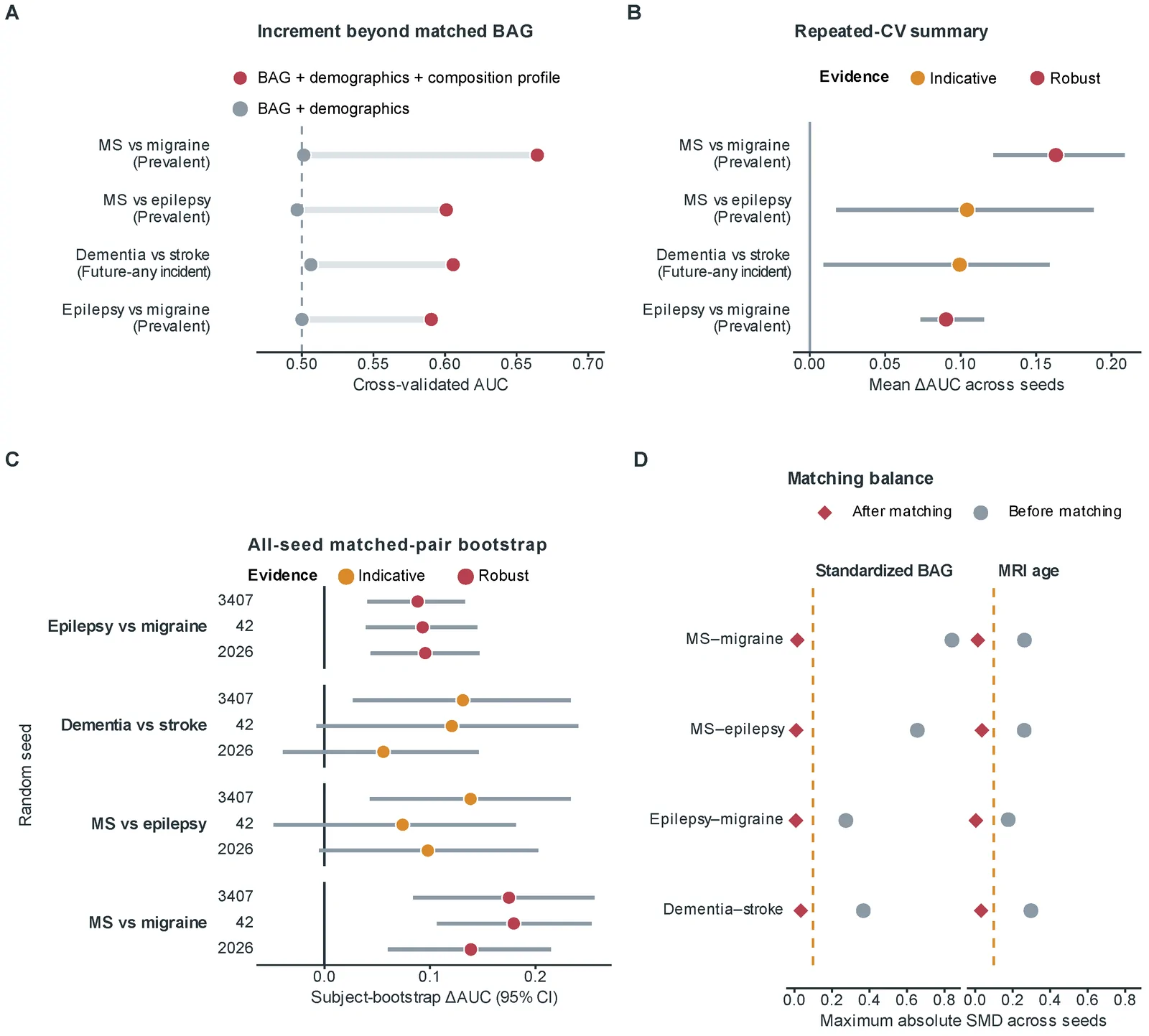
Brain age gap (BAG)-matched disease discrimination using brain age composition profiles. (**A**) Area under the receiver operating characteristic curve (AUC) for the baseline and composition models, evaluated by cross-validation (CV). The composition model additionally included the participant-specific brain age composition profile defined by the global and routed expert representations. (**B**) Mean AUC gain across model seeds ($\Delta \mathrm{AUC}={\mathrm{AUC}}_{\mathrm{profile}}-{\mathrm{AUC}}_{\mathrm{baseline}}$). (**C**) Matched-pair bootstrap estimates of ΔAUC with 95% confidence intervals (CIs). Circles indicate seed-specific ΔAUC estimates, horizontal lines indicate 95% CIs, and vertical black lines mark ΔAUC = 0; orange and red denote indicative and robust evidence, respectively. (**D**) Covariate balance before and after matching, summarized by the maximum absolute standardized mean difference (SMD) in magnetic resonance imaging (MRI) age and standardized BAG. MS denotes multiple sclerosis.

We next assessed whether the profile gains persisted when the disease groups were restricted to participants with a similar MRI age and standardized BAG. After matching, the maximum absolute standardized mean difference in these variables was 0.036 across the four contrasts and three seeds (Figure 5D). The baseline AUCs ranged from 0.497 to 0.506, indicating little discrimination from the balanced scalar covariates.

Adding the profile increased the mean AUC in all four matched contrasts (Table 4 and Figure 5A,B). The largest gain occurred for prevalent multiple sclerosis versus migraine, with the AUC increasing from 0.501 to 0.665 (+0.163). For prevalent epilepsy versus migraine, the AUC increased from 0.500 to 0.590 (+0.090). Positive gains were also observed for multiple sclerosis versus epilepsy (+0.104) and future-any dementia versus stroke (+0.099). These results supported the retained disease-discriminative information among participants with similar overall brain age deviations.

Matched-pair bootstrap analyses assessed support for these gains across model seeds (Figure 5C). Multiple sclerosis versus migraine and epilepsy versus migraine met the robustness criterion, with 95% intervals above zero in all three seeds. Multiple sclerosis versus epilepsy and future-any dementia versus stroke were classified as indicative, with intervals including zero in at least one seed. The latter contrasts contained only 47–53 matched pairs per seed and had less consistent bootstrap support. Because seed-specific matching retained different participant sets, the variation could reflect both model initialization and sample selection.

**Figure 6:**
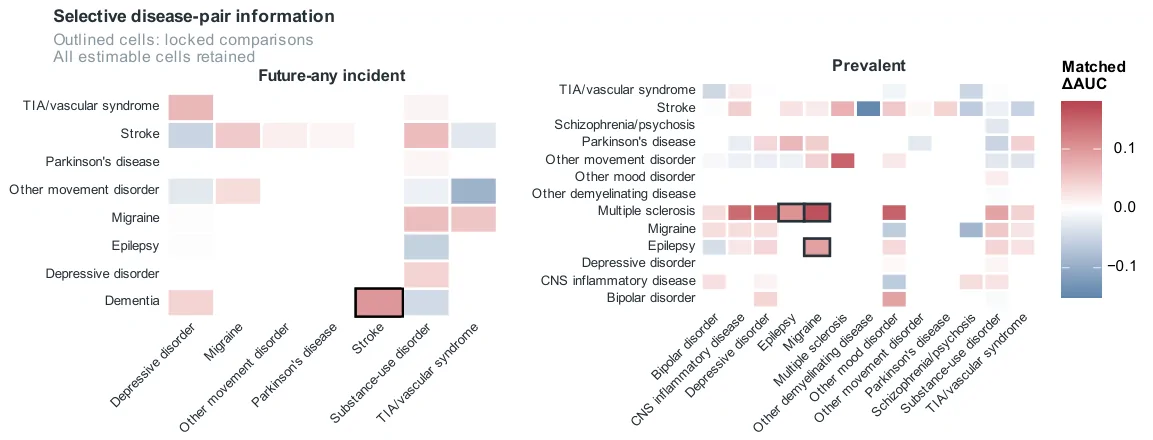
Selectivity of composition profile discrimination across disease pair analyses. Mean profile-versus-baseline ΔAUC is shown for estimable same-phase disease pairs in the prevalent (**left**) and future-any (**right**) analyses. Participants were matched by MRI age and standardized BAG within sex and imaging center strata. Outlined cells indicate the four highlighted contrasts, and blank cells denote non-estimable comparisons. Near-zero and negative estimates are retained.

The complete matched disease pair matrices showed that added discrimination was selective (Figure 6). Other contrasts had positive, near-zero, or negative mean gains, and some could not be estimated. The four highlighted comparisons therefore illustrated retained profile information in selected disease contrasts, while the broader results bounded its applicability across diseases.

## 4. Discussion

This study showed that brain age composition profiles captured disease-related imaging differences beyond the standardized BAG. The semantic-guided mixture-of-experts model produced both an age estimate and a profile of its global and local expert outputs. Participants with a similar standardized BAG could have different profiles, and adding the profile improved discrimination in the four disease contrasts. The profile thus provides additional information about the imaging patterns associated with an overall brain age estimate.

The standardized BAG provides a single measure of the deviation between the predicted brain age and chronological age after age bias correction. Its broad disease associations were consistent with earlier brain age studies [5,6,7]. However, a single BAG value does not specify which imaging characteristics underlie the deviation. Previous studies have addressed this heterogeneity through modality-specific and subsystem-level age estimates [13,21], as well as regional estimates and neuroanatomical ageing dimensions [22,23]. Our framework extends this approach by learning global and local expert outputs jointly within an age prediction model. Retaining these outputs makes it possible to compare imaging patterns among participants with similar overall brain age deviations.

The control comparisons suggest that adding more dimensions alone does not explain the profile’s gains (Table 3). The profile had higher AUC point estimates than PCA-11, Bottleneck-11, and Random-MoE-11 in all four contrasts, although each representation contained 11 features. These controls assessed different aspects of the representation: general imaging variation, age prediction without expert decomposition, and expert grouping without semantic guidance. Metadata on modality, anatomy, and phenotype guided the proposed expert grouping, which was stable across model seeds. The results support the value of this organization under the tested conditions. The profile was not uniformly superior: PCA-22 slightly exceeded it for dementia versus stroke. The reported AUC rankings also do not establish statistically significant differences between representations.

Combining the profile with PCA-11 improved discrimination further. PCA captures the dominant variation in the imaging features without using age labels, whereas the profile is learned through age prediction. Neither representation was trained using disease labels. Their combination achieved the highest AUC in all four contrasts and exceeded PCA-22, which had the same total dimensionality. This pattern suggests that the profile and PCA retain complementary information relevant to disease discrimination. The different learning objectives help to explain this complementarity but do not imply that PCA captures only information unrelated to age.

The matched analysis strengthened the evidence for profile information beyond the standardized BAG (Figure 5). The profile gains persisted when the disease groups had a similar MRI age and standardized BAG, showing that useful imaging differences remained after these variables were balanced. Bootstrap support varied across contrasts, and the broader disease pair analyses showed that the gains were selective. These findings support disease-related imaging differences but do not establish distinct biological ageing mechanisms. The tasks evaluated discrimination between disease groups, so the results cannot establish screening performance or prospective individual risk prediction. Clinical interpretation therefore requires caution, consistent with broader assessments of brain age biomarkers [4,24].

Several limitations constrain the findings. The evaluation was internal to UK Biobank participants aged 44–83 years, without independent validation in other populations. Recorded diagnoses may miss subclinical disease, and diagnosis dates may differ from biological onset. Comparisons across diagnostic phases therefore do not describe within-person ageing trajectories. Small matched groups and differences in participant selection across seeds limit precision. The profiles also depend on the predefined IDPs, and their relationships with cognitive decline, functional outcomes, and longitudinal brain changes were not directly evaluated.

## 5. Conclusions

This study developed a semantic-guided mixture-of-experts framework for individualized brain age composition profiling from multimodal IDPs. In addition to brain age estimation and the standardized BAG, the framework retained a participant-specific representation of the global and expert-specific imaging patterns underlying each prediction. Whereas the standardized BAG summarizes the overall magnitude of brain age deviation, the composition profile captures heterogeneity in the imaging configuration associated with the deviation. These profiles improved disease discrimination beyond the MRI age, standardized BAG, sex, and imaging center in the four highlighted contrasts, with the gains persisting after BAG matching. These findings support brain age composition profiling as a complementary representation for investigating neuroimaging heterogeneity beyond the scalar BAG.

## Figures and Tables

**Table 1:** Representation controls for disease discrimination.

Representation	Added Dim.	Role
Baseline	0	Covariates and standardized BAG only
PCA-11	11	Unsupervised dimensionality reduction control
PCA-22	22	Unsupervised dimensionality reduction control
Bottleneck-11	11	Age-supervised representation control
Random-MoE-11	11	Expert decomposition without semantic organization
Profile	11	Semantically organized expert profile
Profile + PCA-11	22	Semantically organized expert profile + unsupervised dimensionality reduction control

“Added dim.” denotes the number of imaging-derived features added to the common baseline. Abbreviations: BAG, brain age gap; IDP, imaging-derived phenotype; PCA, principal component analysis; MoE, mixture of experts.

**Table 2:** Baseline characteristics of the analysis cohorts.

Analysis Role	Use in Analysis	*N*	Age (Years), Mean (SD)	Age Range (Years)	Female, *N* (%)
Training set	Brain age model fitting and BAG calibration	17,341	63.8 (7.6)	44–82	8867 (51.1)
Validation set	Brain age model selection	3062	63.9 (7.6)	46–82	1568 (51.2)
Test set	Age prediction performance evaluation	3601	63.8 (7.6)	47–82	1842 (51.2)
Clinical evaluation cohort	Disease-associated BAG and composition profile analyses	13,890	64.3 (7.9)	45–83	7951 (57.2)
IDP-QC excluded	Excluded from all analyses after IDP quality control	650	67.7 (7.8)	47–82	242 (37.2)

Values are *N*, mean (SD), range, or *N* (%). The training, validation, and test sets formed the reference cohort for brain age modeling. Participants had no qualifying records for the prespecified conditions at or before MRI or during available follow-up. Other diseases did not preclude inclusion. Brain age model fitting and BAG calibration used the training set only. The clinical evaluation cohort included participants with prevalent or incident qualifying diagnoses and was reserved for downstream disease analyses. All analysis cohorts included four MRI centers. Abbreviations: *N*, number of participants; SD, standard deviation; MRI, magnetic resonance imaging; IDP, imaging-derived phenotype; QC, quality control; BAG, brain age gap.

**Table 3:** Disease discrimination using baseline, composition profile, and control representations.

Representation	MS vs. Migraine (Prevalent)	MS vs. Epilepsy (Prevalent)	Dementia vs. Stroke (Future-Any)	Epilepsy vs. Migraine (Prevalent)
Baseline	0.709	0.689	0.613	0.633
PCA-11	0.739	0.704	0.659	0.641
PCA-22	0.741	0.704	0.676	0.645
Bottleneck-11	0.711	0.689	0.613	0.633
Random-MoE-11	0.719	0.687	0.664	0.631
Profile	0.759	0.723	0.673	0.651
Profile + PCA-11	**0.787**	**0.766**	**0.703**	**0.669**

Values are AUCs rounded to three decimal places. The baseline included MRI age, standardized BAG, sex, and imaging center. Each other classifier added the indicated representation to this baseline. Profile denotes the complete 11-dimensional global and local expert profile; Profile + PCA-11 concatenates this profile with 11 principal components. Bold values indicate the highest reported AUC in each contrast, whereas underlined values indicate the second-highest AUC. Abbreviations: AUC, area under the receiver operating characteristic curve; BAG, brain age gap; MRI, magnetic resonance imaging; MS, multiple sclerosis; PCA, principal component analysis; MoE, mixture of experts.

**Table 4:** BAG-matched disease discrimination with baseline and composition profile classifiers.

Measure	MS vs. Migraine (Prevalent)	MS vs. Epilepsy (Prevalent)	Dementia vs. Stroke (Future-Any)	Epilepsy vs. Migraine (Prevalent)
Matched pairs	87–92	47–50	51–53	227–231
Baseline AUC	0.501	0.497	0.506	0.500
Profile AUC	0.665	0.601	0.606	0.590
$\Delta {\mathrm{AUC}}_{\mathrm{profile},\mathrm{baseline}}$	0.163	0.104	0.099	0.090
Profile gain evidence	Robust	Indicative	Indicative	Robust

Matched-pair counts are ranges across three model seeds; AUCs and ΔAUC values are means across seeds. Robust indicates a 95% bootstrap interval above zero in all three seeds, whereas indicative indicates a positive mean ΔAUC with the interval crossing zero in at least one seed. Abbreviations: BAG, brain age gap; AUC, area under the receiver operating characteristic curve; MS, multiple sclerosis.

## Data Availability

Individual-level UK Biobank data cannot be publicly shared under the terms of the UK Biobank Access and Use Agreement. These data are available to eligible researchers through application to the UK Biobank. This study was conducted using the UK Biobank Resource under application number 46872. The source code is publicly available at https://github.com/wilmerwang/semanticMoE (accessed on 4 October 2026).

## Abbreviations

ARI	Adjusted Rand index
AUC	Area under the receiver operating characteristic curve
BAG	Brain age gap
CI	Confidence interval
CV	Cross-validation
FLAIR	Fluid-attenuated inversion recovery
FDR	False discovery rate
ICD-10	International Classification of Diseases, 10th Revision
IDP	Imaging-derived phenotype
MAE	Mean absolute error
MLP	Multilayer perceptron
MoE	Mixture of experts
MRI	Magnetic resonance imaging
MS	Multiple sclerosis
NMI	Normalized mutual information
PCA	Principal component analysis
QC	Quality control
REC	Research Ethics Committee
SD	Standard deviation
SMD	Standardized mean difference

## Appendix A

**Table A1:** Reference cohort disease exclusion criteria.

ICD-10 Codes	Exclusion Category	Included Conditions or Examples
F00–F03	Dementia	Dementia in Alzheimer’s disease, vascular dementia, dementia in other diseases, and unspecified dementia
I60–I69	Cerebrovascular diseases	Intracranial haemorrhage, cerebral infarction, arterial occlusion or stenosis, other cerebrovascular diseases, and their sequelae
G00–G09	Inflammatory diseases of the central nervous system	Meningitis, encephalitis, myelitis, intracranial or intraspinal abscess, and related conditions
G10–G13	Systemic atrophies primarily affecting the central nervous system	Huntington’s disease, hereditary ataxia, spinal muscular atrophy and related syndromes, and systemic atrophies in other diseases
G20–G26	Extrapyramidal and movement disorders	Parkinson’s disease, secondary parkinsonism, dystonia, and other movement disorders
G30–G32	Other degenerative diseases of the nervous system	Alzheimer’s disease and other degenerative nervous system disorders
G35–G37	Demyelinating diseases of the central nervous system	Multiple sclerosis, other acute disseminated demyelination, and other central nervous system demyelinating diseases
G40–G41	Epilepsy and status epilepticus	Epilepsy and status epilepticus
G43–G44	Migraine and other headache syndromes	Migraine and other headache syndromes
G45–G46	Transient cerebral ischaemic attacks and vascular syndromes	Transient cerebral ischaemic attacks and related syndromes and vascular syndromes of the brain in cerebrovascular diseases
G80–G83	Cerebral palsy and other paralytic syndromes	Cerebral palsy, hemiplegia, paraplegia, tetraplegia, and other paralytic syndromes
G91–G94	Hydrocephalus and other brain disorders	Hydrocephalus, toxic encephalopathy, and other brain disorders, including those in diseases classified elsewhere
F10–F19	Substance-related mental and behavioral disorders	Mental and behavioral disorders due to psychoactive substance use
F20–F29	Schizophrenia, schizotypal, and delusional disorders	Schizophrenia, schizotypal disorder, delusional disorders, and other psychotic disorders
F30–F39	Mood (affective) disorders	Manic episodes, bipolar disorder, depressive disorders, and other mood disorders

Codes denote three-character ICD-10 categories and include their subcategories. These criteria define exclusion from the reference cohort and are broader than the disease categories used for clinical evaluation. Participants with no qualifying records at or before MRI or during available follow-up were eligible for the reference cohort. Other diagnoses did not preclude eligibility unless they met one of the listed exclusion criteria.

## Appendix B

We assessed imaging center transfer by developing the semantic-guided mixture-of-experts model using reference cohort participants from center 11025 only. Participant-level quality control used the missing IDP criterion described in the main text. Preprocessing parameters were estimated from source-center training participants and applied unchanged to the validation and evaluation participants. The model used ten local experts and one global expert. Models were trained separately for seeds 2026, 42, and 3407 without reusing the main analysis checkpoints. Source-center validation participants were used for model selection. Centers 11026–11028 were excluded from training, preprocessing fitting, and model selection.

The primary evaluation used the original reference-test participants at each center (Table A2). A secondary evaluation included all eligible reference participants at the unseen centers, including those previously assigned to the main study’s training or validation sets (Table A3). These participants were not used to develop the source-only models. The secondary samples included the reference-test participants at each target center, so the evaluations overlap. The MAE, RMSE, and mean signed prediction error (predicted brain age minus chronological age) were calculated separately for each seed and averaged across seeds. Percentile 95% confidence intervals were estimated from 2000 participant bootstrap samples within each center, using identical participant indices across seeds. The intervals are conditional on the trained models and do not include model refitting. Seed-specific results are provided in Table A4.

**Table A2:** Single-center model development and reference-test prediction performance.

A. Sample Sizes				
**Center ID**	**Role**	**Training N**	**Validation N**	**Test N**
11025	Source center	10,026	1706	2055
11026	Unseen center	0	0	585
11027	Unseen center	0	0	953
11028	Unseen center	0	0	8
**B. Mean Test Performance Across Three Model Seeds**				
**Center ID**	**MAE (95% CI)**	**RMSE (95% CI)**	**Bias (95% CI)**	
11025	2.994 (2.902, 3.091)	3.785 (3.674, 3.900)	−0.046 (−0.203, 0.118)	
11026	2.941 (2.765, 3.135)	3.768 (3.553, 4.007)	−0.766 (−1.066, −0.470)	
11027	2.858 (2.739, 2.984)	3.567 (3.426, 3.712)	−0.440 (−0.656, −0.210)	
11028	2.024 (0.962, 3.336)	2.734 (1.218, 3.926)	−0.658 (−2.558, 0.983)	

All errors are expressed in years. Bias denotes mean predicted brain age minus chronological age. Source-center test participants were excluded from training and model selection; target centers were entirely excluded from model development. Center 11028 had only eight test participants, so its results are descriptive. Abbreviations: *N*, number of participants; MAE, mean absolute error; RMSE, root mean squared error; CI, confidence interval.

**Table A3:** Secondary evaluation in all eligible reference participants at unseen centers.

Center ID	*N*	MAE (95% CI)	RMSE (95% CI)	Bias (95% CI)
11026	4020	2.963 (2.895, 3.029)	3.750 (3.669, 3.830)	−0.641 (−0.747, −0.531)
11027	6165	2.893 (2.842, 2.944)	3.643 (3.580, 3.704)	−0.425 (−0.511, −0.338)
11028	32	3.022 (2.188, 3.985)	4.027 (2.857, 5.163)	−0.410 (−1.753, 0.923)

Values are arithmetic means across three seeds with participant-bootstrap 95% CIs. All errors are expressed in years. These samples include the reference-test participants at each target center and are not independent of the primary evaluation. None was used to train or select the source-only models.

The source-center test MAE was 2.994 years. At the two larger unseen centers, the test MAE was 2.941 years (center 11026) and 2.858 years (center 11027). The mean signed errors were negative at both centers, with 95% intervals below zero, indicating systematic age underprediction. The smallest center had only eight reference-test and 32 secondary evaluation participants, limiting precision. This experiment evaluates transfer from one source center within UK Biobank, rather than four-fold leave-one-center-out or external cohort validation. It cannot separate scanner effects from differences in demographic composition or training sample size or establish generalizability to arbitrary scanners and acquisition protocols.

**Table A4:** Seed-specific prediction performance of the source-center-only models.

Evaluation	Center ID	Seed	*N*	MAE	RMSE	Bias
Reference test	11025	2026	2055	3.008	3.799	−0.218
Reference test	11025	42	2055	2.991	3.788	−0.057
Reference test	11025	3407	2055	2.982	3.768	0.138
Reference test	11026	2026	585	3.001	3.835	−0.993
Reference test	11026	42	585	2.948	3.787	−0.827
Reference test	11026	3407	585	2.875	3.681	−0.479
Reference test	11027	2026	953	2.908	3.621	−0.626
Reference test	11027	42	953	2.853	3.570	−0.594
Reference test	11027	3407	953	2.815	3.510	−0.099
Reference test	11028	2026	8	2.022	2.748	−0.666
Reference test	11028	42	8	2.307	3.084	−1.202
Reference test	11028	3407	8	1.743	2.370	−0.105
Expanded set	11026	2026	4020	3.001	3.795	−0.912
Expanded set	11026	42	4020	3.002	3.793	−0.710
Expanded set	11026	3407	4020	2.886	3.663	−0.302
Expanded set	11027	2026	6165	2.919	3.680	−0.615
Expanded set	11027	42	6165	2.926	3.688	−0.558
Expanded set	11027	3407	6165	2.833	3.562	−0.101
Expanded set	11028	2026	32	2.866	3.901	−0.607
Expanded set	11028	42	32	3.226	4.159	−0.530
Expanded set	11028	3407	32	2.973	4.019	−0.094

All errors are expressed in years. “Expanded set” denotes the secondary evaluation in all eligible reference participants at unseen centers. Seeds use the same participants and do not represent independent cohorts. Reference-test and expanded samples overlap at each target center.
